# Mechanical Properties of Polyurethane Mixture and Load Response Behaviour of Polyurethane Composite Pavement

**DOI:** 10.3390/polym15020417

**Published:** 2023-01-12

**Authors:** Wei Zhuang, Yufeng Bi, Baoju Liu, Derui Hou, Shuo Jing, Xiaojin Lu, Min Sun

**Affiliations:** 1 School of Civil Engineering, Central South University, Changsha 410075, China; 2 Shandong Provincial Communications Planning and Design Institute Co., Ltd., Jinan 250031, China; 3 School of Transportation Engineering, Shandong Jianzhu University, Jinan 250101, China

**Keywords:** mechanical properties, polyurethane mixture, load response, composite pavement, pavement structure

## Abstract

Finite element numerical simulation calculation of pavement structure load response is widely applied; however, there is still a lack of research on the polyurethane (PU) mixture composite pavement load response. The mechanical characteristics of PU mixture composite pavement are not well understood, and there is a lack of research on typical pavement structures of PU mixtures, which limits their application in pavement structures. Therefore, herein, the mechanical properties of PU mixtures are analysed using the dynamic modulus test, uniaxial penetration test, and fatigue test. Further, the finite element theory calculation method is used to realize the load response calculation of orthogonal design composite pavement structure. The results show that PU mixtures exhibit more obvious elastic characteristics and have good shear resistance, fatigue stability, and temperature stability, and can be used as shear and anti-fatigue layers. The structure of ‘4 cm SMA-13 + 5 cm PUM-20 + 6 cm PUM-25 + semi-rigid base’ is recommended for the PU mixture composite structure. In comparison to typical asphalt pavement, the analysis shows that except for shear stress, temperature has little effect on the load response of PU composite pavement structures, while high temperatures lead to a significant increase in the load response of typical asphalt pavement structures. The PU composite pavement can bear greater loads and has a reduced thickness of its surface layer by about 3 cm in comparison to conventional pavement. The results of this study provide theoretical support for the design of PU mixture pavement structures and promote the popularization and application of PU mixture pavement.

## 1. Introduction

Asphalt pavement has the properties of good driving comfort, low noise during driving, strong environmental adaptability, and technical and economic benefits, so it is widely used in all kinds of road pavement [1]. At present, the pavement structure of three-layer surfaces and cement-stabilized gravel bases is often adopted in China [2]. However, as a viscoelastic discontinuous material, the mechanical properties of asphalt mixtures are easily affected by external environmental factors. Early issues such as ruts, potholes, cracks, and uneven subsidence occur frequently during the use of asphalt mixtures [3]. Much fuel is consumed to heat mineral materials and asphalt; this produces large amounts of emissions [4]. The aforementioned defects of asphalt mixtures have become an important factor restricting the higher-quality development of pavement [5].

At present, there is an increasing body of research on polyurethane (PU) mixtures in pavement engineering [6,7,8,9,10]. Compared to traditional mixtures, PU mixtures have better durability, temperature stability, waterproof properties, and crack resistance, which can reduce maintenance frequency [11,12,13,14,15]. However, as a new type of road construction material, the conventional pavement structure design method is not applicable [16,17,18,19,20]. Therefore, it is necessary to study the mechanical properties of PU mixtures and their composite pavement load response behaviour, providing a basis for the structural design of PU mixture pavement.

At present, some research has been conducted into the load response of composite pavement structures. Angel Mateos et al. identified the two main distress mechanisms in concrete overlays on asphalt pavement based on the analysis of the structural response of test sections and finite element modelling [21]. A two-dimensional finite element model was developed using the Mich-Pave software to predict pavement responses, and the responses of flexible pavement were evaluated comprehensively [22]. A three-dimensional finite element (3D FE) model was used by Feng et al. to investigate the temperature field and thermal responses; it was found that the maximum principal tensile stresses of the contact interface between the pipe and the surrounding concrete were larger [23]. Haibin Wei used the ANSYS static structural analysis module to analyse three indicators of EPDM pavement [24]. Zhu et al. used a finite element method to analyse the mechanical response of hydronic asphalt pavement (HAP); the results indicated that the concrete between adjacent pipes was subjected to significant tensile stresses [25]. The mechanical response of hydronic asphalt pavement under temperature–vehicle coupled loading was evaluated by Zhu et al., who found that the concrete between adjacent pipes was subjected to significant tensile stresses [25]. Huang et al. established a numerical model to characterize the fracture process of a reinforced concrete (RC) beam strengthened with fiber-reinforced polymer (FRP) in detail [26]. Yu et al. carried out an investigation on marble, and an impact factor of weak disturbance was defined [27]. Li et al. proposed a transfer learning pipeline and enabled a distress detection model to be applied to other untrained scenarios [28]. Different models, such as finite element models [29], three-dimensional finite element models [30], and ANSYS static structure modules [31], have been adopted by scholars to assess and analyse pavement structural responses, composite pavement, and snow-melting heated bridge deck systems [32,33].

It is clear that the current calculation method of pavement structure load response is relatively mature; however, there is a lack of research on PU mixture composite pavement load responses. The mechanical characteristics of PU mixture composite pavement are not known, and there are no relevant suggestions for the typical pavement structure of PU mixtures, which limits the popularization of PU mixtures in pavement structures. Therefore, the load responses of orthogonal design composite pavement structures are analysed basing on mechanical properties of PU mixtures and numerical simulation calculations, and the PU mixture composite pavement structure is recommended. The load response of PU mixture composite pavement structures is comprehensively analysed and compared with that of typical asphalt pavement structures, which provides theoretical support for the design of PU mixture pavement structures and promotes the popularization and application of PU mixture green roads.

## 2. Material Composition and Typical Pavement Structure

### 2.1. Raw Materials

(1) Polyurethane binder

In this study, a one-component moisture-curable PU binder is used, produced by Wanhua Chemical Co., Ltd., Yantai, China. It is a modified isocyanate prepolymer containing a certain terminal isocyanate (NCO) group, which is polymerized by isocyanate, polyether polyol, vegetable oil, and catalysts. The specific technical indicators of 25 °C are shown in Table 1.

The NCO group in the PU binder is extremely chemically active. The first step of the reaction is that the NCO group reacts with water to form diamines and releases carbon dioxide. The second step is that the diamine substance further reacts with NCO, undergoes chain extension reactions, and generates the urea group, which solidifies the material.

(2) Asphalt

The 70# base asphalt and SBS-modified asphalt are used, the technical indicators of which meet the provisions of JTG F40-2004 [35].

### 2.2. Mixture Composition

**Figure 1:**
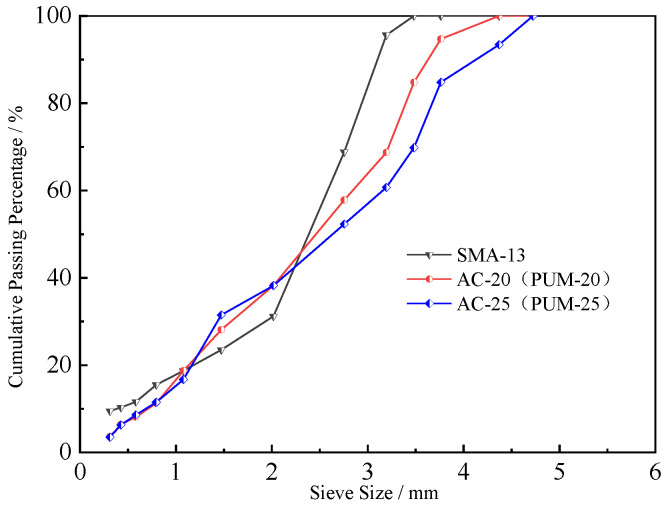
Mineral aggregate gradation of the mixture.

The composition design of SBS-modified asphalt with a maximum nominal particle size of 13.2 mm (SMA-13), PU mixture, and asphalt mixture with a maximum nominal particle size of 19.0 mm and 26.5 mm (PUM-20, AC-20, PUM-25, AC-25) are carried out respectively. The aggregate gradations of the mixtures are shown in Figure 1. The composition design results of the mixtures are shown in Table 2.

## 3. Test Scheme and Calculation Theory

### 3.1. Test Scheme

#### 3.1.1. Dynamic Modulus Test

The dynamic modulus specimens are cylinders with a diameter of 100 mm and height of 150 mm, and one set of tests has three parallel specimens. According to the pavement working temperature, four temperatures of 10 °C, 20 °C, 35 °C, and 50 °C are selected, and nine loading frequencies of 0.1 Hz, 0.2 Hz, 0.5 Hz, 1.0 Hz, 2.0 Hz, 5.0 Hz, 10 Hz, 20 Hz, and 25 Hz are used. The specimens are kept at the test temperature for 5 h before tested. The dynamic modulus test is carried out in accordance with T 0738-2011 of JTG E20-2011 [36]. The five mixtures’ dynamic modulus tests are carried out.

#### 3.1.2. Uniaxial Penetration Test

According to JTG D50-2017, uniaxial penetration tests of the five mixtures are conducted [37]. The UTM-100 dynamic hydraulic test system is used for the test. A cylinder with a diameter of 42 mm is used. The loading rate is 1 mm/min and the pressure and displacement are recorded. When the stress value is reduced to 90% of the stress extreme point, the test is stopped, and the maximum shear failure load is taken at the inflexion point of the test curve, accurate to 0.001 KN.

**Figure 2:**
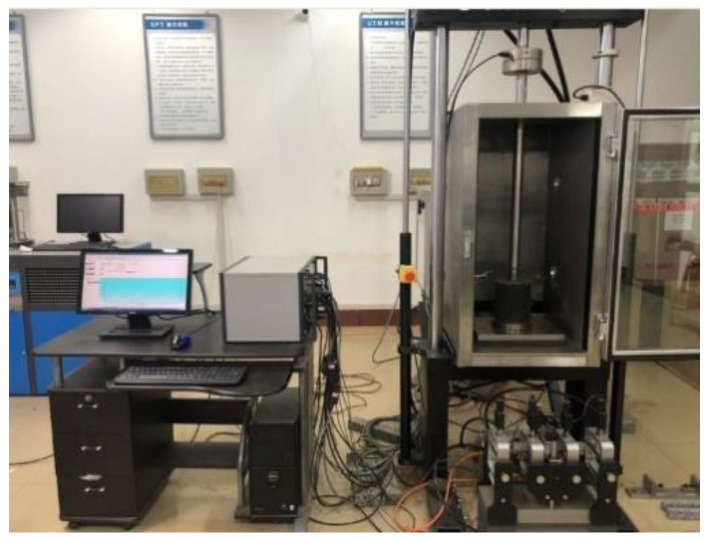
Uniaxial penetration test device.

The uniaxial penetration tests of the five mixtures are carried out at 15 °C, 20 °C, 30 °C, 40 °C, 50 °C, and 60 °C. The uniaxial penetration strength and modulus of the mixture are calculated as follows. The test apparatus and loading method are shown in Figure 2.

#### 3.1.3. Four-Point Bending Fatigue Test

Firstly, four-point bending static loading tests of five mixtures were carried out. The specimen size is 380 mm × 50 mm × 63.5 mm and the test temperature is 25 °C ± 0.5 °C. The displacement control mode is used to load at a speed of 0.01 mm/s until the specimen is broken, and the ultimate bearing capacity and flexural strength are obtained [32].

Secondly, according to the ultimate bearing capacity and stress level, the control stresses of the five mixtures are calculated. Previous studies have shown that the fatigue life of PU mixtures is long [16,17], so the stress levels of asphalt mixtures are 0.3, 0.4, and 0.5, and the stress levels of PU mixtures are 0.5, 0.6, and 0.7.

**Figure 3:**
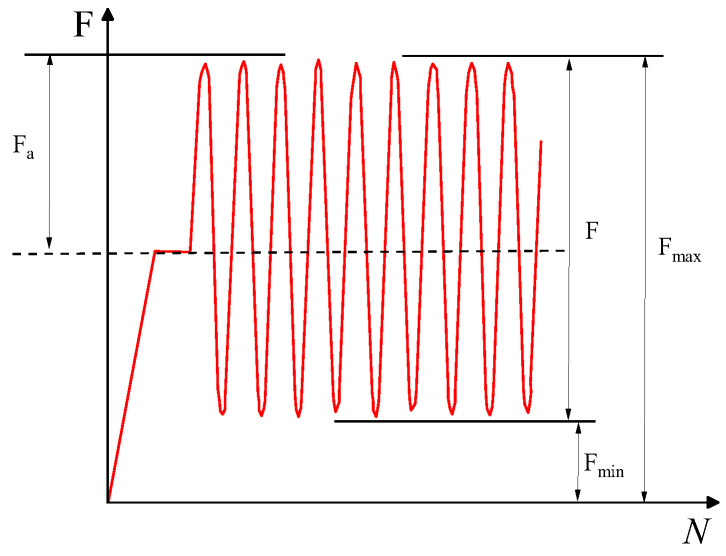
Loading waveform curve.

**Figure 4:**
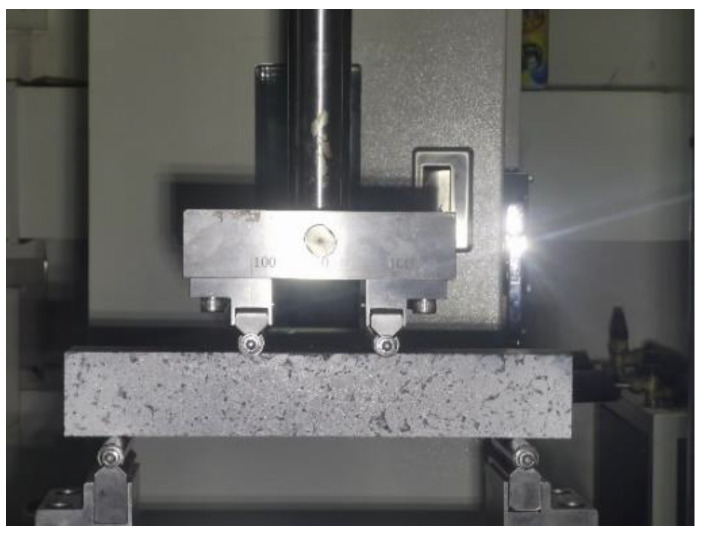
Specimen loading method.

Finally, four-point bending fatigue tests are carried out under stress control mode. Continuous partial sinusoidal loading mode, loading frequency 10 Hz ± 0.1 Hz, loading waveform is a non-interrupted asymmetric constant amplitude sine wave. The cyclic characteristic value (the ratio of F_min_ to F_max_) is R = 0.1, where the maximum cyclic loading is the product of the stress level S and the ultimate bearing capacity, and the minimum loading is the product of the maximum value and the cyclic characteristic value R [33]. Load is not stopped until fatigue fracture of the specimen occurs. The loading waveform curve is shown in Figure 3, and the loading method is shown in Figure 4.

### 3.2. Orthogonal Design of PU Mixture Composite Pavement Structure

Three-layer surfaces and cement-stabilized gravel bases are often adopted in China, and the uppermost layer is usually an SBS-modified asphalt mixture wear layer to maintain the pavement [13,14,15]. Therefore, the 4 cm SMA-13 mixture is used as the upper layer of the pavement, and this study focuses on the structural design of the middle and lower layers. Previous studies have shown that PU mixtures have significant elastic properties, and their dynamic modulus is obviously larger [16,17]. Therefore, the thickness of the PU mixture layers is appropriately reduced in the design of the pavement structure.

The four-factor mixed-level orthogonal design of composite pavement structures is shown in Table 3. The middle and lower layers are given different thicknesses: the middle layer thicknesses are 3 cm, 4 cm, and 5 cm; the lower layer thicknesses are 6 cm, 7 cm, and 8 cm. The AC-20 and PUM-20 mixtures are randomly matched to the middle layer, the AC-25 and PUM-25 mixtures are randomly matched to the lower layer, and the base layer is a double-layer cement stabilized gravel mixture.

The deflection of the pavement surface, vertical compressive strain of the asphalt surface, shear stress between the asphalt layer and PU layer, tensile stress at the bottom of the base layer, and vertical compressive strain at the top of the subgrade are selected as key mechanical calculation indexes of the pavement load response. In addition, the material cost of the pavement structure is one of the key factors to be considered in pavement structure design. The pavement material cost calculation results of pavement with a width of 30 m and length of 100 m are used as an evaluation index for orthogonal design.

### 3.3. Theoretical Calculation

#### 3.3.1. Finite Element

**Figure 5:**
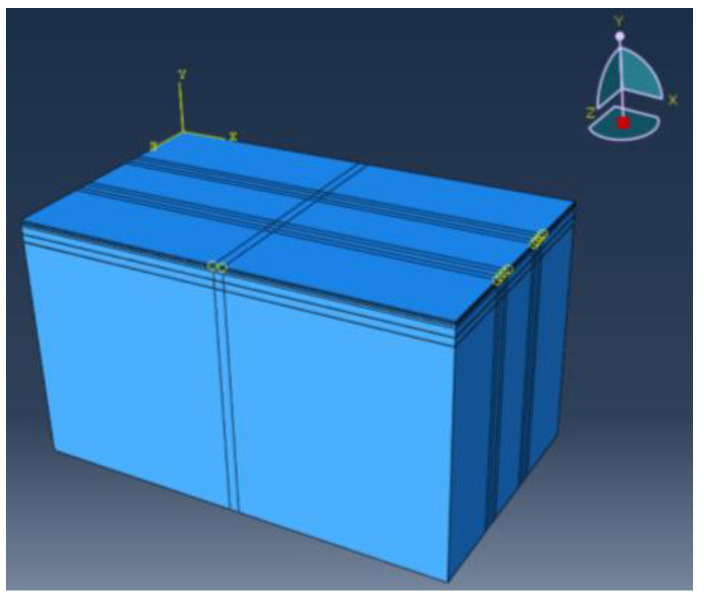
Finite element for the pavement structure.

The load responses of PU mixture composite pavement are calculated using the ABAQUS finite element software. The model size is 10 m (length) × 6 m (width) × 6 m (height); the x-direction in the model is the driving direction, the y-direction is the road depth direction, and the z-direction is the lateral direction of the road. The standard axle load of BZZ-100 is selected for model loading, the tire grounding pressure P is 0.7 MPa, and the tire grounding size is 0.226 m × 0.156 m. The elastic continuous layered system theory is used for calculation [34]. The structural layers of the pavement are connected through binding. The materials of each layer are divided by eight-node linear hexahedral elements. The established model structure is shown in Figure 5.

#### 3.3.2. Calculation Parameters and Conditions

The calculation models for the twelve pavement structures in the four-factor mixed-level orthogonal design table are established. Based on the analysis of the load responses of orthogonal design pavement structure, the PU mixture composite pavement structure is recommended. The load responses of the PU mixture composite pavement structure and typical asphalt pavement structure, with temperatures of 20 °C and 50 °C, are calculated and compared. The material parameters of different materials are shown in Table 4.

The vertical compressive strain of the asphalt surface layer, tensile stress of the base bottom, road surface deflection, maximum shear stress, and vertical compressive strain of the subgrade surface are taken as the orthogonal design analysis calculation indexes.

## 4. Results and Discussion

### 4.1. Dynamic Modulus Test Results

#### 4.1.1. Dynamic Modulus

**Figure 6:**
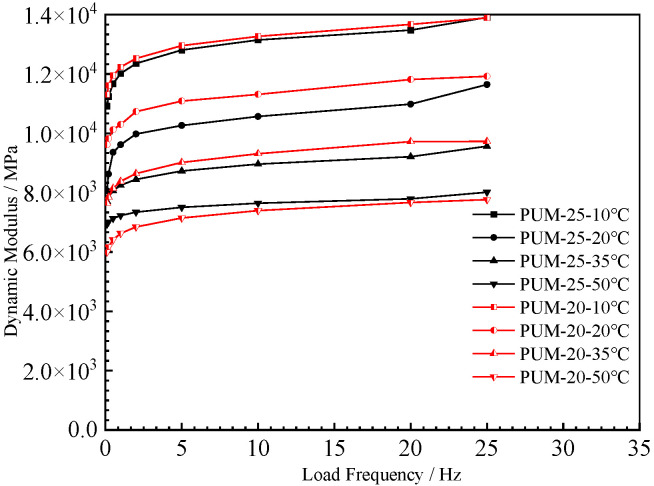
Dynamic modulus of PU mixtures.

**Figure 7:**
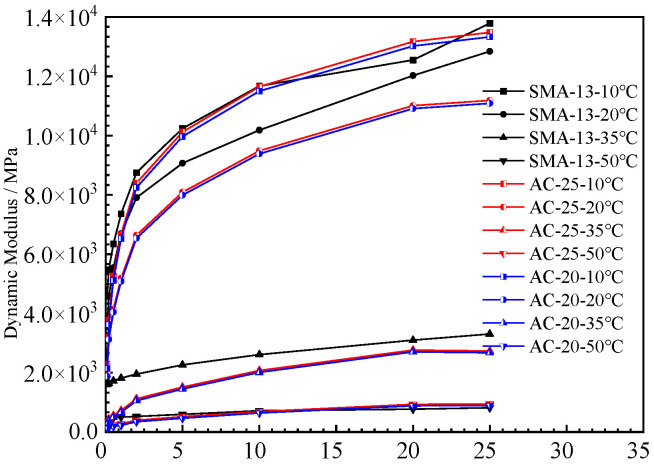
Dynamic modulus of asphalt mixtures.

The dynamic modulus test results for the five mixtures are shown in Figure 6 and Figure 7. The results show that the higher the test temperature, the lower the dynamic modulus of the five mixtures at the same loading frequency. The high temperature reduces the ability of the five mixtures to resist loading. With increases in the test temperature, the dynamic modulus of the SMA-13, AC-20, and AC-25 mixtures decrease greatly, while those of the PUM-20 and PUM-25 mixtures decrease slightly. This indicates that SMA-13, AC-20, and AC-25 mixtures are significantly affected by temperature, while the PUM-20 and PUM-25 mixtures have better temperature stability.

The dynamic modulus of the two mixtures increased with increases in the loading frequency. The dynamic modulus of the SMA-13, AC-20, and AC-25 mixtures is obviously affected by the loading frequency. The dynamic modulus of PUM-20 and PUM-25 mixtures at different loading frequencies changes slightly, and the influence of different loading frequencies on PU mixtures is relatively small.

The reason for this is that asphalt is a temperature-sensitive binder. Increases in the temperature lead to a transition of asphalt from an elastic state to a viscous state, which affects its mechanical properties [16]. The strength of the PU mixture is formed by the curing reaction of the PU binder, which is irreversible. When the ambient temperature rises, the mechanical properties of PU mixtures do not change; thus, PU mixtures exhibit good temperature stability.

#### 4.1.2. Phase Angle

**Figure 8:**
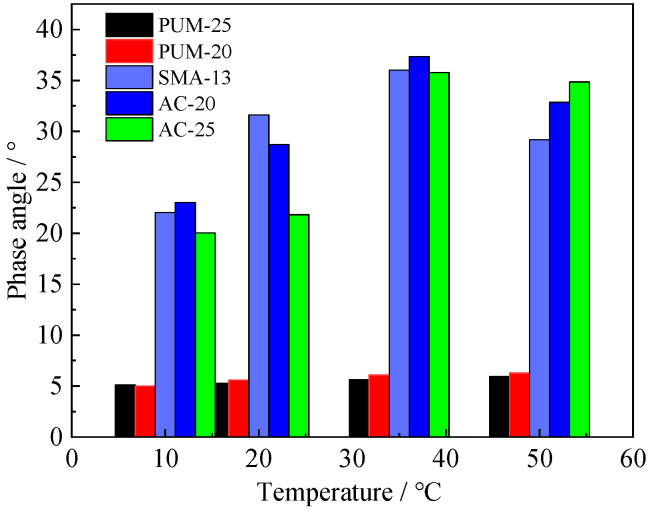
Phase angle at 10 Hz loading frequency.

**Figure 9:**
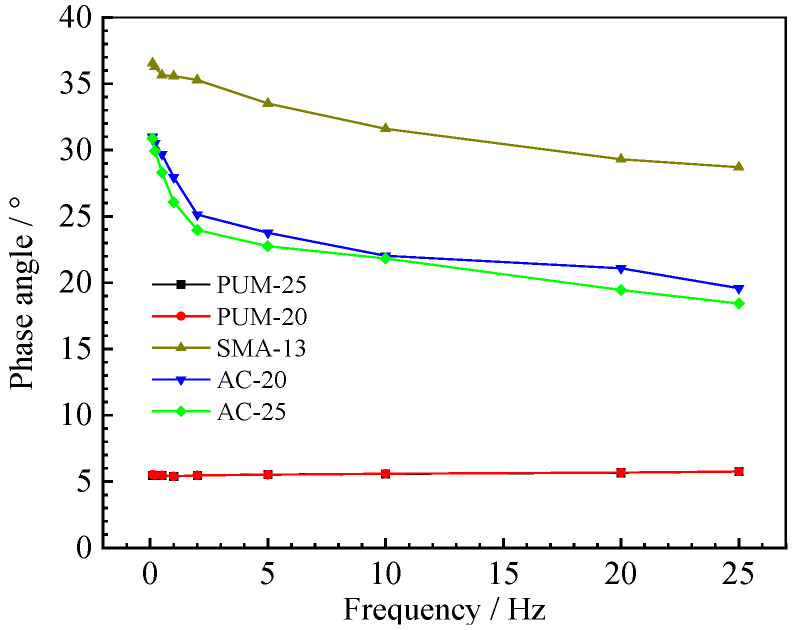
Phase angle at 20 °C.

The phase angle of 10 Hz is shown in Figure 8, and the phase angle of 20 °C is shown in Figure 9.

Under different test temperatures and loading frequencies, the phase angles of PUM-20 and PUM-25 mixtures change between 4° and 6°; the overall change range is within 2°. The phase angles of SMA-13, AC-20, and AC-25 mixtures are between 15–40°, and the phase angle varies greatly with the loading frequency and test temperature. The phase angle is used as an index to analyse the elasticity and viscosity coefficient of the material. The larger the phase angle, the more obvious the viscosity characteristics of the material. The test results show that the elastic properties of PU mixtures are relatively obvious, while the viscous properties of the asphalt mixture are relatively obvious; further, the temperature has little effect on the viscoelastic properties of PU mixtures.

### 4.2. Uniaxial Penetration Test Results

**Figure 10:**
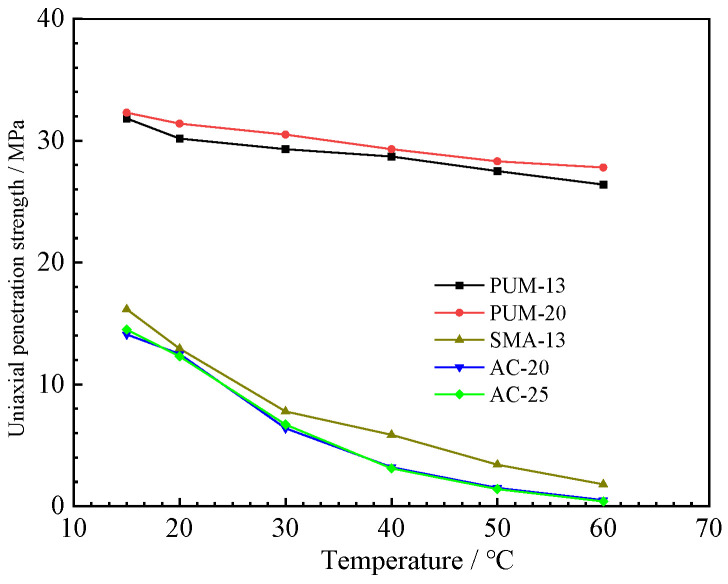
Uniaxial penetration strength results.

**Figure 11:**
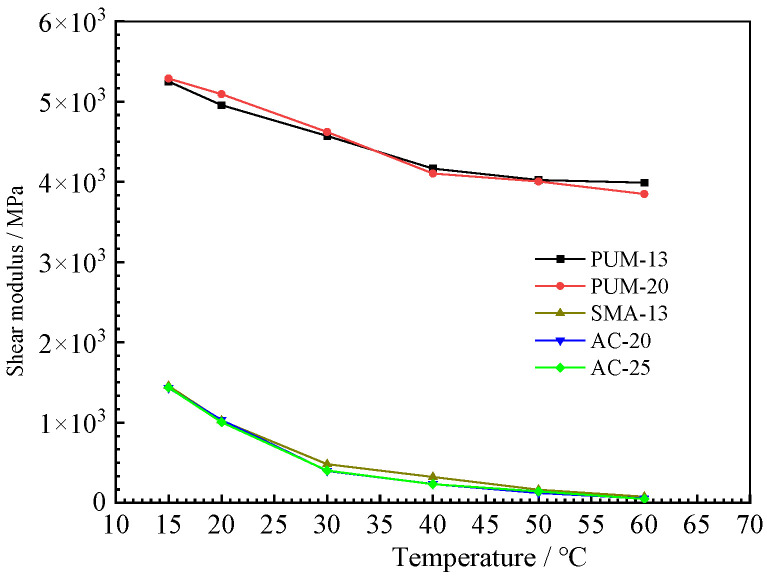
Shear modulus test results.

The uniaxial penetration strength and shear modulus of the five mixtures are shown in Figure 10 and Figure 11. The uniaxial penetration strength and shear modulus of PUM-20 and PUM-25 mixtures are far greater than those of SMA-13, AC-20, and AC-25 mixtures, and the temperature has little influence on the uniaxial penetration strength of PU mixtures [8]. The PU mixtures have high uniaxial penetration strengths and are less affected by temperature, indicating their high bearing capacity and good temperature stability. The shear moduli of PUM-20 and PUM-25 mixtures are much larger than those of SMA-13, AC-20, and AC-25 mixtures, and the temperature has little effect on the shear modulus of PU mixtures, which is consistent with the dynamic modulus test results. Therefore, PU mixtures can be applied to the shear layer of pavement structures.

### 4.3. Four-Point Bending Fatigue Test Results

#### 4.3.1. Four-Point Bending Static Loading Tests

**Figure 12:**
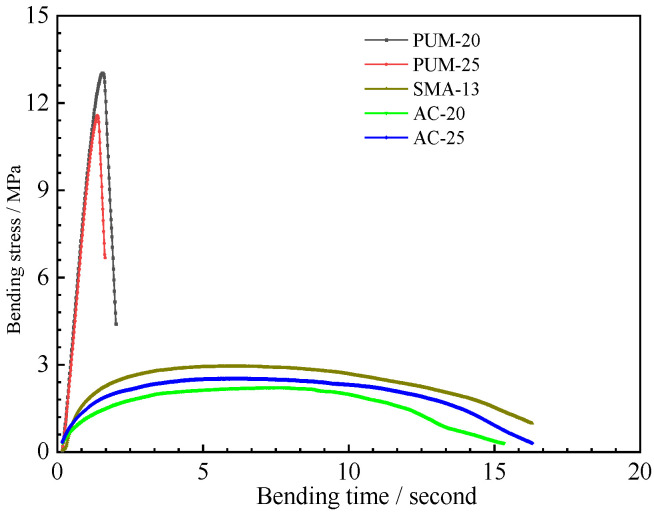
Time–stress curve.

**Figure 13:**
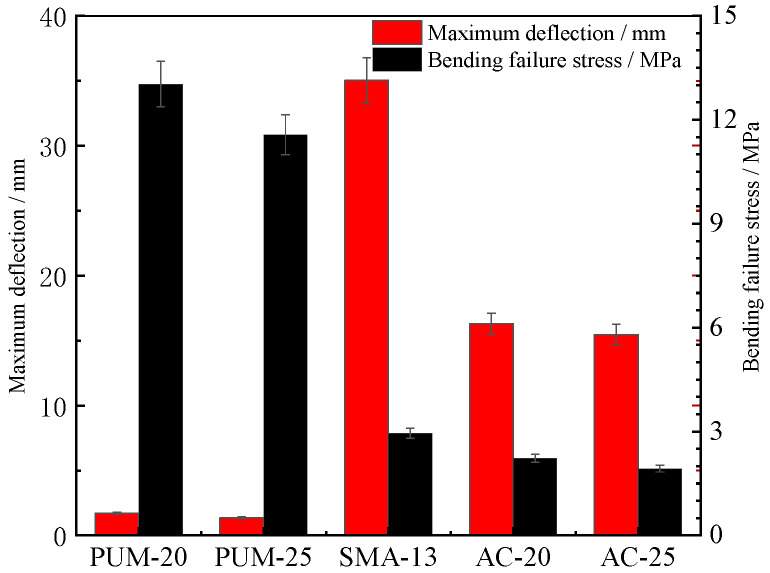
Maximum deformation and damage loads.

The four-point bending load–displacement curves of the five mixtures are shown in Figure 12. The maximum deformation and bending failure stress are shown in Figure 13.

The load–displacement curves of the PUM-20 and PUM-25 mixtures differ from those of the SMA-13, AC-20, and AC-25 mixtures. The bending stresses of PUM-20 and PUM-25 mixtures increase rapidly under loading, and the mixture fails after bending for about 3 s, while the time–stress curves of PUM-20 and PUM-25 mixtures are relatively flat, and the mixture fails after loading for 15 s. In addition, the bending failure stress of PUM-20 and PUM-25 is about four times larger than that of SMA-13, AC-20, and AC-25, while the maximum deformation of SMA-13, AC-20, and AC-25 is about 15 times larger than that of PUM-20 and PUM-25. This indicates that the PU mixture has a strong ability to resist loading; however, its plastic deformation under loading is small, easily leading to brittle failure [12].

#### 4.3.2. Four-Point Bending Fatigue Tests

**Figure 14:**
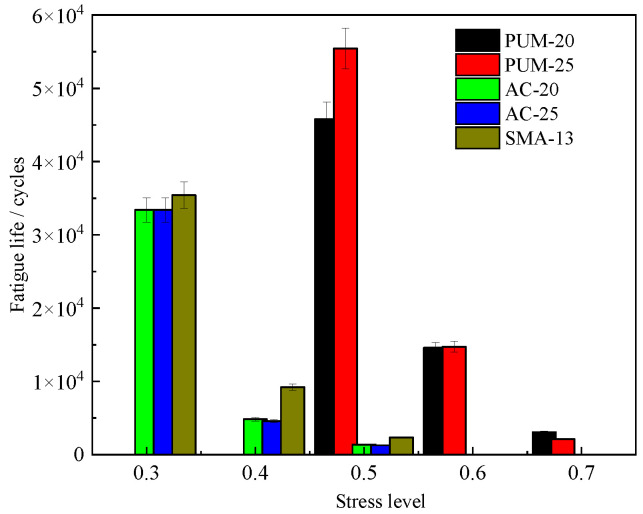
Fatigue life of mixtures.

The stress-controlled four-point bending fatigue life test results of the five mixtures are shown in Figure 14.

With increases in the stress level, the fatigue life of the five mixtures gradually decreases. When the stress level of SMA-13, AC-20, and AC-25 mixtures increases to 0.5, the fatigue life is only 1000–2000 cycles, while the fatigue life of the PUM-20 and PUM-25 mixtures at the 0.7 stress level can still reach 2000–3000 cycles; that is, PU mixtures still have good fatigue resistance at high stress levels. At the 0.5 stress level, the fatigue life of PUM-20 and PUM-25 mixtures is more than 25 times that of the SMA-13, AC-20, and AC-25 mixtures, indicating that the PU mixture has a strong ability to resist fatigue load [12]. The four-point bending fatigue test shows that the PU mixture has strong fatigue resistance and can be applied at higher stress levels.

### 4.4. Analysis of Pavement Structure Orthogonal Design Results and Pavement Structure Recommendation

The calculation results for the twelve pavement structures of the orthogonal design table are shown in Table 5.

The range (ΔK) under different factors and levels is calculated as shown in Table 6, and is then used to analyse the impacts of each factor on different indicators in the orthogonal test, and to determine the optimal factor combination corresponding to different indicators.

According to the results of the aforementioned method, the influence factors for the mechanical indexes of pavement structures are discussed as follows. The factors affecting the vertical compressive strain of the surface layer and the tensile stress of the base bottom are ranked as A > B > C = D. The pavement structure with A3B3C2D1 can obtain the minimum vertical compressive strain of the asphalt surface layer and the tensile stress at the bottom of the base layer. The influence order of the pavement surface deflection factors is C = D > B > A. The pavement structure of C2D2B3A3 can obtain the minimum pavement surface deflection and improve the pavement-bearing capacity. The influence of the maximum shear stress factors is B > A > C = D, and the B3A3C1D2 pavement structure can obtain the minimum interlayer shear stress and reduce the interlayer displacement. The influence of vertical compressive strain factors on the top surface of the subgrade is A > B > C = D, and the compressive strain of the top surface of the A3B3C1D2 pavement structure is the smallest, reducing the deformation of the subgrade. The influence of the material cost factors is B > A > C = D, and the pavement structure cost of B1A1 is the lowest.

Combining various load responses and pavement structure costs, the final preferred pavement structure is A3B1C2D2; that is, ‘4 cm SMA + 5 cm PUM-20 + 6 cm PUM-25′ is proposed as the PU and asphalt mixture composite structure.

### 4.5. Load Response Behaviour of Composite Pavement Structures

**Figure 15:**
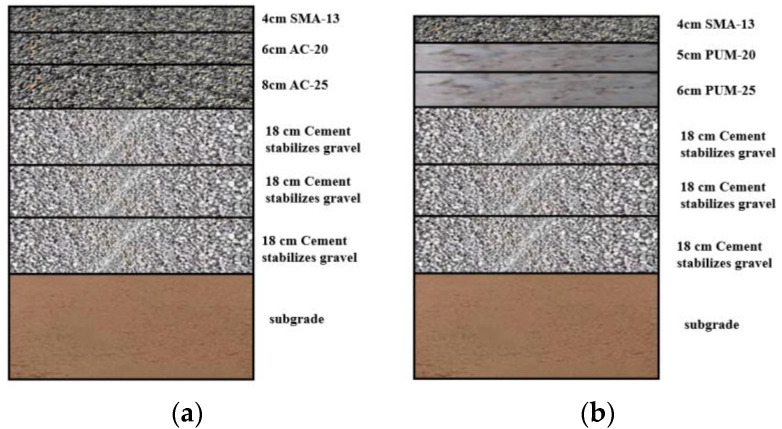
Schematic diagram of the two pavement structures. (**a**) Typical pavement structure. (**b**) Composite pavement structure.

To comprehensively characterize the load response of PU composite pavement, the load responses of typical asphalt pavement structure (typical structure) and PU and asphalt mixture composite pavement (composite structure) are also calculated and compared. The two pavement structures are shown in Figure 15.

#### 4.5.1. Vertical Compressive Strain of the Subgrade Surface

**Figure 16:**
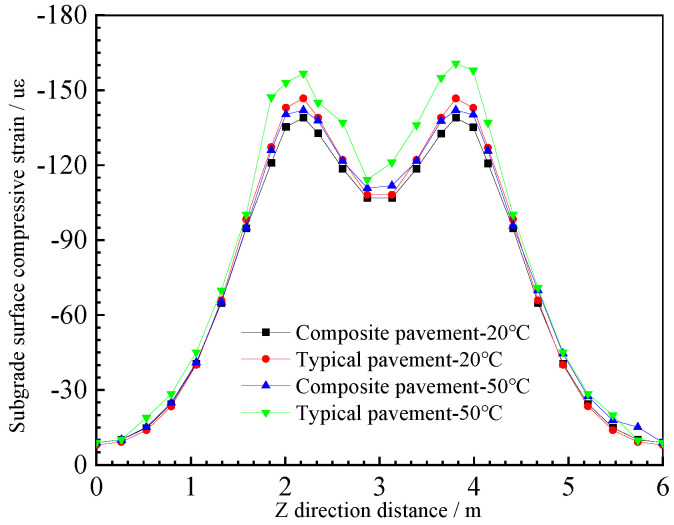
Subgrade surface compressive strain.

The vertical compressive strain calculation results of the subgrade surface are shown in Figure 16.

It can be seen that the load response curves of PU and asphalt composite pavement and typical asphalt mixture pavement are similar. The vertical compressive strain of the top surface of the subgrade decreases with increases in the load distance. With increases in the temperature, the subgrade surface compressive strain of the two pavement structures increases, and the temperature has a great influence on the subgrade surface compressive strain of typical asphalt mixture pavement. The maximum subgrade surface compressive strain changes from 142 µε to 167 µε, while the temperature has a relatively small influence on the subgrade surface compressive strain of the PU composite pavement; the maximum subgrade surface compressive strain changes from 139 µε to 142 µε. This may be because the PU mixture has good temperature stability, and the mechanical properties change slightly under high-temperature conditions, while the asphalt mixture changes from elastic to viscous under high-temperature conditions, resulting in large vertical compressive strains on the top surface of the subgrade of typical asphalt pavement [30,38].

#### 4.5.2. Interlayer Shear Stress

**Figure 17:**
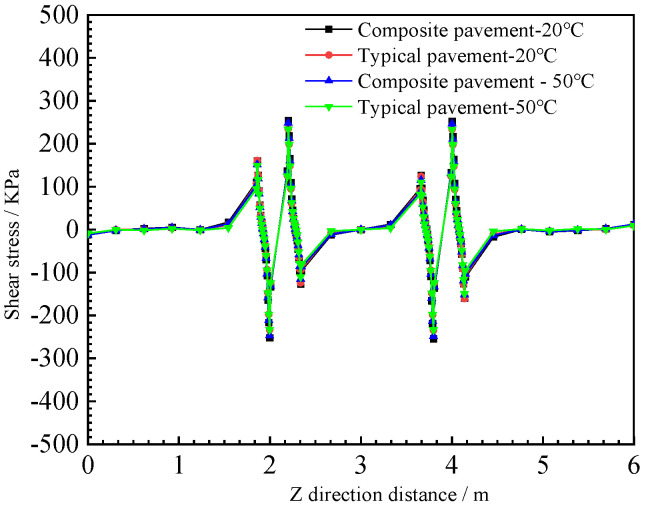
Upper-layer maximum shear stress.

**Figure 18:**
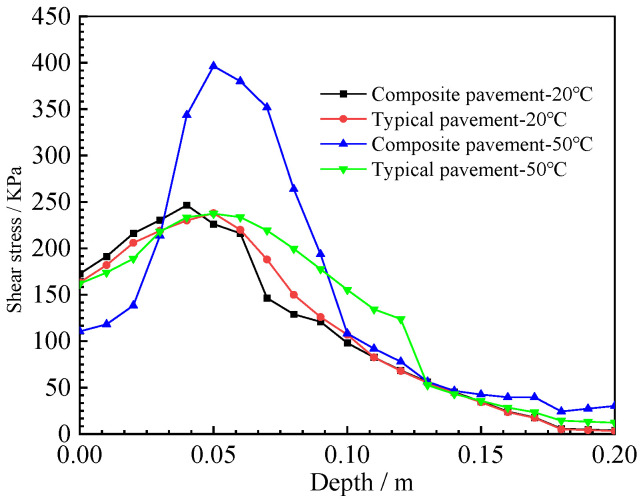
Shear stress in the depth.

The upper-layer bottom shear stress and the maximum shear stress in the depth direction of the two pavement structures are shown in Figure 17 and Figure 18.

The maximum shear stress at the bottom of the upper layer decreases with increases in the load distance, as shown in Figure 17. The maximum shear stress at the bottom of the upper layer of the PU composite pavement and the typical asphalt pavement structure at 10 °C and 50 °C is similar to the lateral distribution of the road. The temperature has little effect on the upper-layer maximum shear stress of the typical asphalt pavement structure, except the wheel load position. The interlayer shear stress of the composite pavement load position under high-temperature conditions reaches 254 KPa.

The shear stress distribution curve in the depth direction of the load position is shown in Figure 18. The shear stress value is larger at the depth of 4–10 cm of the two pavement structures. The shear stress distribution in the depth direction of typical asphalt pavement structures is only slightly affected by temperature changes; however, the shear stress of the PU mixture composite pavement structure increases at high temperatures, and the shear stress value reaches 400 KPa at a 4–6 cm depth of the pavement structure [31]. Therefore, it is necessary to carry out layer treatments between the PU mixture and asphalt mixture layer; on the basis of previous research results, a two-component PU adhesive layer of 0.4 L/m^2^ should be sprayed.

#### 4.5.3. Vertical Compressive Strain of the Composite Pavement Structure Surface

**Figure 19:**
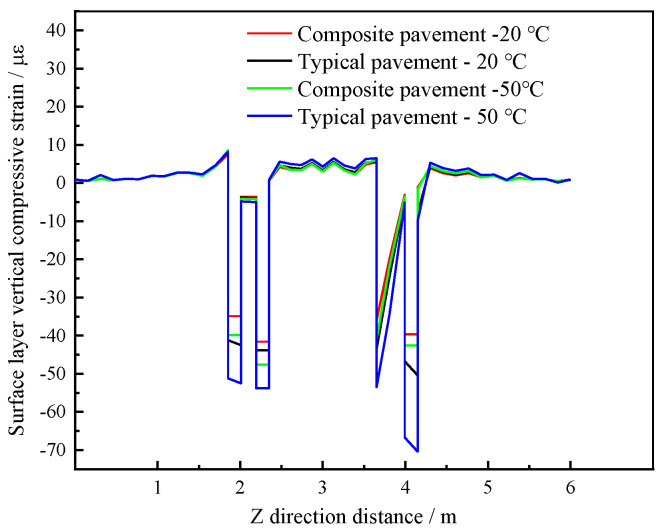
Vertical compressive strain of the surface layer.

The vertical compressive strain curves of the surface layer in Figure 19 show that the two structures exhibit the same change trends. The vertical compressive strain of the PU mixture composite pavement is relatively small at different temperatures, and the values are close, indicating that the pavement structure has high-temperature stability, mainly due to the strength formed by the cross-linking curing reaction of the PU mixture, which does not easily deform with temperature changes [32,39]. However, the vertical compressive strain of typical asphalt pavement is the largest at 50 °C, primarily due to the high-temperature sensitivity of the asphalt binder.

#### 4.5.4. Deflection

**Figure 20:**
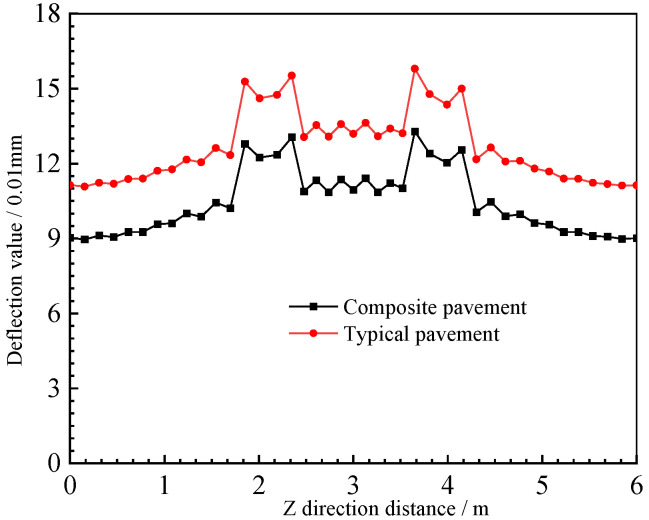
Deflections of the two pavement structures.

The deflection calculation results of the two pavement structures are shown in Figure 20. It can be seen that the maximum deflection appears at the position of load action, and the deflection of the composite pavement structure is smaller than that of typical asphalt pavement, which indicates that PU mixture composite pavement has a higher structural bearing capacity and can be adapted to higher-grade loads.

#### 4.5.5. Composite Pavement Structure Base-Layer Bottom Tensile Stress

**Figure 21:**
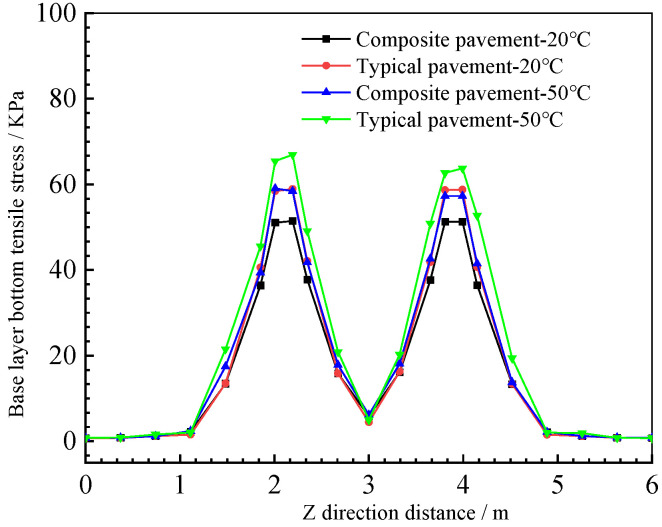
Bottom tensile stress of the base layer.

From the base-layer bottom tensile stress curves in Figure 21, it can be seen that the change trends of the two structures are the same; the stress decreases with increases in the load distance, and the base-layer bottom tensile stress at the same position increases with increases in the temperature. Further, the temperature has a great influence on the base-layer bottom tensile stress of typical asphalt pavement, and the base-layer bottom tensile stress of asphalt pavement under high-temperature conditions is the largest. Because of the lower base-layer bottom tensile stress, a relatively long service life of the PU composite pavement base is ensured [40].

In conclusion, the responses of the two pavement structures near the edge of the pavement are relatively similar, but the mechanical response values of the PU and asphalt composite pavement structures near the load are relatively small; in addition to the shear stress, the temperature has little effect on the load responses of the PU composite pavement structure, and high-temperature effects lead to significant increases in the load response of the typical asphalt pavement. This shows that the PU and asphalt composite pavement can bear greater loads and resist higher temperatures, and the surface layer thickness of PU and asphalt composite pavement is about 3 cm smaller than that of typical asphalt pavement, which can save a significant amount of road construction material. Furthermore, the excellent durability of the PU mixture can effectively reduce the maintenance and reconstruction cost investment, reducing the interference of road traffic flow during operation.

## 5. Conclusions

The PU mixtures exhibit more obvious elastic characteristics and have good shear resistance, fatigue stability, and temperature stability, and can be used as shear and anti-fatigue layers; the conclusions are as follows:

(1) The influence of the temperature and loading frequency on the dynamic modulus and phase angle of PU mixtures is relatively small. The phase angle is between 4° and 6°, and the elastic characteristics of the PU mixture are relatively stable. The uniaxial penetration strength and shear modulus of the PU mixture are greater than those of asphalt mixtures, and are less affected by temperature. PU mixtures have good shear resistance and temperature stability, and can be used as shear layers.
(2) The bending failure stress of PU mixtures is much larger than that of asphalt mixtures, which have a strong ability to resist loads; however, its plastic deformation is small under loading, and the failure speed is fast, which may be more prone to brittle failure. PU mixtures have excellent fatigue resistance and can be applied to anti-fatigue layers due to the need for use in layers with high stress levels.
(3) Based on the calculation of load responses of orthogonal design pavement structures, the typical PU mixture composite pavement structure is recommended, which is a ‘4 cm SMA-13 + 5 cm PUM-20 + 6 cm PUM-25 + semi-rigid base’ pavement structure.
(4) In comparison to the load response calculation structure of typical asphalt pavement, the analysis shows that—other than for shear stress—temperature has little effect on the load response of PU composite pavement structure. The PU composite pavement can bear greater loads and has strong resistance to high temperatures. The load responses of PU composite can meet the requirements of high-grade highways, heavy load pavement, long longitudinal slope sections, and intersection sections.
(5) The theory of elastic layered system is adopted. The viscoelastic characteristics of asphalt mixture are not fully considered, which may affect the numerical analysis results to a certain extent. The recommended polyurethane mixture composite pavement structure is still more expensive than conventional asphalt pavement structure, and can potentially be applied to special road sections.

## Figures and Tables

**Table 1:** Technical indexes of PU binder [34].

Technology Index	Unit	Technical Requirement	Typical Values
Viscosity	mPa·s	1200–2200	1600
Isocyanate group content	%	10.3–11.3	11.1
Density	g/cm^3^	1.05–1.11	1.08
Surface dry time	h	5–10	6
Tensile strength	MPa	≥10	26
Elongation at break	%	≥100	200

**Table 2:** The composition design results for various mixtures.

Mixture	Binder	Nominal Maximum Particle Sizes/mm	Binder Content/%	Void Ratio (VV)
SMA-13	SBS-modified asphalt	13.2	5.8	5.3
AC-20	70# based asphalt	19.0	5.0	4.5
AC-25	70# based asphalt	26.5	4.8	4.6
PUM-20	PU binder	19.0	4.8	4.9
PUM-25	PU binder	26.5	4.6	5.0

**Table 3:** Orthogonal test design of PU and asphalt composite pavement.

Numbering	Factor A (Middle Layer Thickness mm)	Factor B (Lower Layer Thickness mm)	Factor C (Middle Layer Material)	Factor D (Lower Layer Material)
1	1 (30 mm)	1 (60 mm)	1 (AC-20)	2 (PUM-25)
2	2 (40 mm)	2 (70 mm)	1	2
3	3 (50 mm)	3 (80 mm)	1	2
4	1	2	1	2
5	2	3	1	2
6	3	1	1	2
7	1	2	2 (PUM-20)	1 (AC-25)
8	2	3	2	1
9	3	1	2	1
10	1	3	2	1
11	2	1	2	1
12	3	2	2	1

**Table 4:** Material parameters of each pavement structure layer.

Structure Layer Type	20 °C Dynamic Modulus/MPa	50 °C Dynamic Modulus/MPa	Poisson Ratio
SMA-13	10,184	720	0.25
AC-20	9383	642	0.25
AC-25	9483	692	0.25
PUM-20	11,310	7395	0.25
PUM-25	10,567	7642	0.25
Cement-stabilized gravel (base)	16,000	16,000	0.25
Cement-stabilized gravel (subbase)	12,000	12,000	0.25
subgrade	70	70	0.40

**Table 5:** Orthogonal test results for PU mixture composite pavement.

Number	Factor A	Factor B	Factor C	Factor D	Vertical Compressive Strain of Asphalt Surface/με	Tensile Stress of Base Bottom/10^−3^ MPa	Road Surface Deflection/0.01 mm	Maximum Shear Stress/10^−3^ Mpa	Vertical Compressive Strain of Subgrade Top/με	Material Cost/CNY Ten Thousand
1	1	1	1	2	52.86	137.5	17.10	49.10	39.6	89.5
2	2	2	1	2	48.49	138.0	26.24	49.80	39.42	97.9
3	3	3	1	2	40.13	126.4	13.00	38.00	36.70	106.4
4	1	2	1	2	53.91	134.6	16.85	48.93	38.75	94.8
5	2	3	1	2	40.24	128.7	13.11	36.40	37.32	103.3
6	3	1	1	2	39.95	132.0	13.37	39.80	38.31	95.7
7	1	2	2	1	52.38	135.1	16.60	49.35	39.05	94.8
8	2	3	2	1	39.60	128.8	13.07	43.60	37.48	103.3
9	3	1	2	1	39.65	130.8	13.18	39.90	38.00	95.7
10	1	3	2	1	39.62	131.8	13.26	43.10	38.37	100.2
11	2	1	2	1	39.62	133.8	13.77	43.40	38.89	92.6
12	3	2	2	1	39.65	128.3	13.03	43.60	37.31	101.0

**Table 6:** Orthogonal data processing results.

Response	Range (ΔK)				Optimal Structure Combination
	Factor A	Factor B	Factor C	Factor D	
Vertical compressive strain of surface layer/με	39.39	34.84	25.06	25.06	A3B3C2D1
Tensile stress of base bottom/10^−3^ MPa	2.15	2.03	0.86	0.86	A3B3C2D1
Pavement surface deflection/0.01 mm	13.61	20.28	41.76	41.76	C2D2B3A3
Maximum shear stress/10^−3^ Mpa	29.18	30.58	0.92	0.92	B3A3C1D2
Vertical compressive strain of subgrade top/με	5.45	4.93	1	1	A3B3C1D2
Material cost/CNY ten thousand	19.5	39.7	0	0	B1A1CD

## Data Availability

The data used to support the findings of this study are available from the corresponding author upon request.
